# Aβ oligomers: role at the synapse

**DOI:** 10.18632/aging.101818

**Published:** 2019-02-08

**Authors:** Daniela Puzzo

**Affiliations:** 1 Department Biomedical and Biotechnological Sciences, University of Catania, Catania 95123, Italy

**Keywords:** Alzheimer’s disease, Amyloid-β peptide, oligomers, synaptic plasticity, memory

For decades, the increase of Amyloid-beta (Aβ) levels has been considered the early event that triggers Alzheimer’s disease (AD). Although several evidences continue supporting the main role of Aβ in AD, therapeutic approaches aimed at decreasing its levels have failed so far, raising several questions on AD pathophysiology and thus dividing the neuroscience community [1]. While many researchers assert that Aβ is definitely the *primum movens* of the disease and the anti-Aβ treatment needs to be started in the earlier phases, i.e. when cognitive impairment is not yet manifested, others argue that the increase of Aβ is not crucial in AD, suggesting that we need to focus on other targets, such as tau protein.

Within this context, it would be useful to take into account a different point of view based on several evidences suggesting that Aβ, prior to be the “AD protein”, is a neuromodulator playing a crucial role at the synapse in physiological conditions [2]. As demonstrated by a number of *in vitro* and *in vivo* pre-clinical studies, Aβ: i) is released at hippocampal synapses where it modulates release probability [3]; ii) is produced during memory induction [4]; iii) is needed for formation of memory and its cellular correlate long-term potentiation (LTP) [4–6]. Furthermore, when administered at low picomolar doses, resembling the physiological content in the brain, Aβ induces a beneficial effect resulting in the enhancement of LTP and memory [7].

Although the Aβ dose-dependent opposite effect might merely confirm that “*sola dosis facit venenum*”, several decades of research in this field make the plot even more intricate. Indeed, different Aβ isoforms with a different propensity to form aggregates are present in the brain. In particular, most of the works have focused on Aβ_42_, known to have a higher tendency to form oligomers, whose increase tightly correlates with synaptic dysfunction. As a consequence, the physiological effect of the peptide has been ascribed to monomeric Aβ and, in the bench-to-bedside approach, this idea has been translated into the discovery of anti-Aβ drugs targeting oligomers but sparing monomers (Patel, 2015).

However, in previous studies from our laboratory and others both the positive and negative effects of the peptide were obtained by using a preparation containing a mixture of Aβ_42_ monomers and oligomers [4–6], suggesting that oligomeric Aβ_42_ is also involved in physiological synaptic function. This is not surprising considering that Aβ is present in the healthy brain in different species ranging from monomers to oligomers [7], and it is unlikely that the latter only represent a waste product aimed at inducing AD.

The physiological function of oligomeric Aβ_42_ has been confirmed by our recent work designed to clarify whether different Aβ concentrations, isoforms and aggregation status influence hippocampal LTP and spatial memory [8]. We found that oligomeric Aβ_42_ produced an opposite response on LTP and memory depending upon the concentration (200 nM vs. 200 pM). On the contrary, monomeric Aβ_42_ impaired LTP and memory when at 200 nM, but did not enhance them at pM concentrations. Furthermore, the depletion of endogenous murine Aβ resulted in a dramatic impairment of LTP and memory that was exclusively rescued by 200 pM oligomeric human Aβ_42_. Interestingly, WB and electron microscopy analysis indicated that both monomers and oligomers were present in our 200 pM and 200 nM preparations, but with a different monomer/oligomer ratio, suggesting that the higher is Aβ_42_ concentration the higher is the formation of oligomers.

In conclusion, our findings suggest that the presence of Aβ_42_ oligomers is crucial either in physiological or pathological conditions. This should prompt the neuroscience community to answer at least two crucial questions: why and how a protein that exerts a physiological function in the healthy brain starts increasing in sporadic AD (where this increase is not genetically-driven)? Is it safe to remove Aβ oligomers from the brain considering their involvement in synaptic function? We believe that an in depth knowledge of the mechanisms underlying Aβ production and function in the healthy brain should be achieved to understand the causes leading to its increase and detrimental effect in the AD brain [2].
